# Supplementary material for: Multimodal monitoring of human cortical organoids implanted in mice reveal functional connection with visual cortex
Source: Nat Commun. 2022 Dec 26;13:7945. doi: 10.1038/s41467-022-35536-3 (PMC9792589; doi:10.1038/s41467-022-35536-3)
Supplement: Supplementary file 1 — Supplementary Information [file 41467_2022_35536_MOESM1_ESM.pdf]

Supplementary Figures for "Multimodal monitoring of human cortical organoids implanted in mice reveal functional connection with visual cortex"

Madison N. Wilson[1,#], Martin Thunemann[2,#], Xin Liu[1], Yichen Lu[1], Francesca Puppo[3], Jason W. Adams[3,4], Jeong-Hoon Kim[1], Mehrdad Ramezani[1], Donald P. Pizzo[5], Srdjan Djurovic[6-9], Ole A. Andreassen[7,9-12], Abed A. Mansour[13,14], Fred H. Gage[13], Alysson R. Muotri[3,4,15-17], Anna Devor[2,18,*], and Duygu Kuzum[1,*]

[1]Department of Electrical and Computer Engineering, University of California San Diego, La Jolla, CA, USA
[2]Department of Biomedical Engineering, Boston University, Boston, MA, USA
[3]Department of Pediatrics, University of California San Diego, School of Medicine, La Jolla, CA, USA.
[4]Department of Cellular and Molecular Medicine, University of California San Diego, School of Medicine, La Jolla, CA, USA.
[5]Department of Pathology, University of California San Diego, La Jolla, CA, USA.
[6]Department of Medical Genetics, Oslo University Hospital, Oslo, Norway.
[7]NORMENT Center, Oslo, Norway.
[8]Department of Clinical Science, University of Bergen, Bergen, Norway.
[9]K. G. Jebsen Center for Neurodevelopmental Disorders, University of Oslo, Oslo, Norway.
[10]Division of Mental Health and Addiction, Oslo University Hospital, Oslo, Norway.
[11]Institute of Clinical Medicine, University of Oslo, Oslo, Norway.
[12]Oslo University Hospital, University of Oslo, Norway.
[13]Laboratory of Genetics, The Salk Institute for Biological Studies, La Jolla, CA, USA
[14]Department of Medical Neurobiology, The Hebrew University of Jerusalem, Ein Kerem-Jerusalem, Israel.
[15]Center for Academic Research and Training in Anthropogeny, University of California San Diego, La Jolla, CA, USA.
[16]Archealization Center, University of California San Diego, La Jolla, CA, USA.
[17]Kavli Institute for Brain and Mind, University of California San Diego, La Jolla, CA, USA.
[18]Athinoula A. Martinos Center for Biomedical Imaging, Department of Radiology, Harvard Medical School, Massachusetts General Hospital, Charlestown, MA, USA

[#] *These authors contributed equally.*
[*]*These authors jointly supervised this work.*

Correspondence:

Dr. Duygu Kuzum
Electrical & Computer Engineering
University of California
San Diego
9500 Gilman Drive MC 0407
La Jolla, CA, USA 92093
Email: dkuzum@eng.ucsd.edu

Dr. Anna Devor
Biomedical Engineering
Boston University
Boston
610 Commonwealth Ave.
Boston, MA, USA 02215
Email: adevor@bu.edu

Graphene microelectrode impedance over time

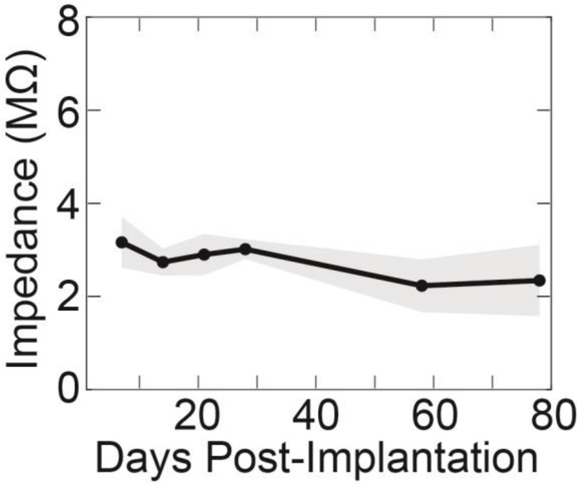

Supplementary Figure 1. Graphene microelectrode impedance over time for a representative animal. Results are shown as mean ± sdv across 16 channels. Channels above 4 M Ω were counted as not working and excluded from analysis.

LFP over time

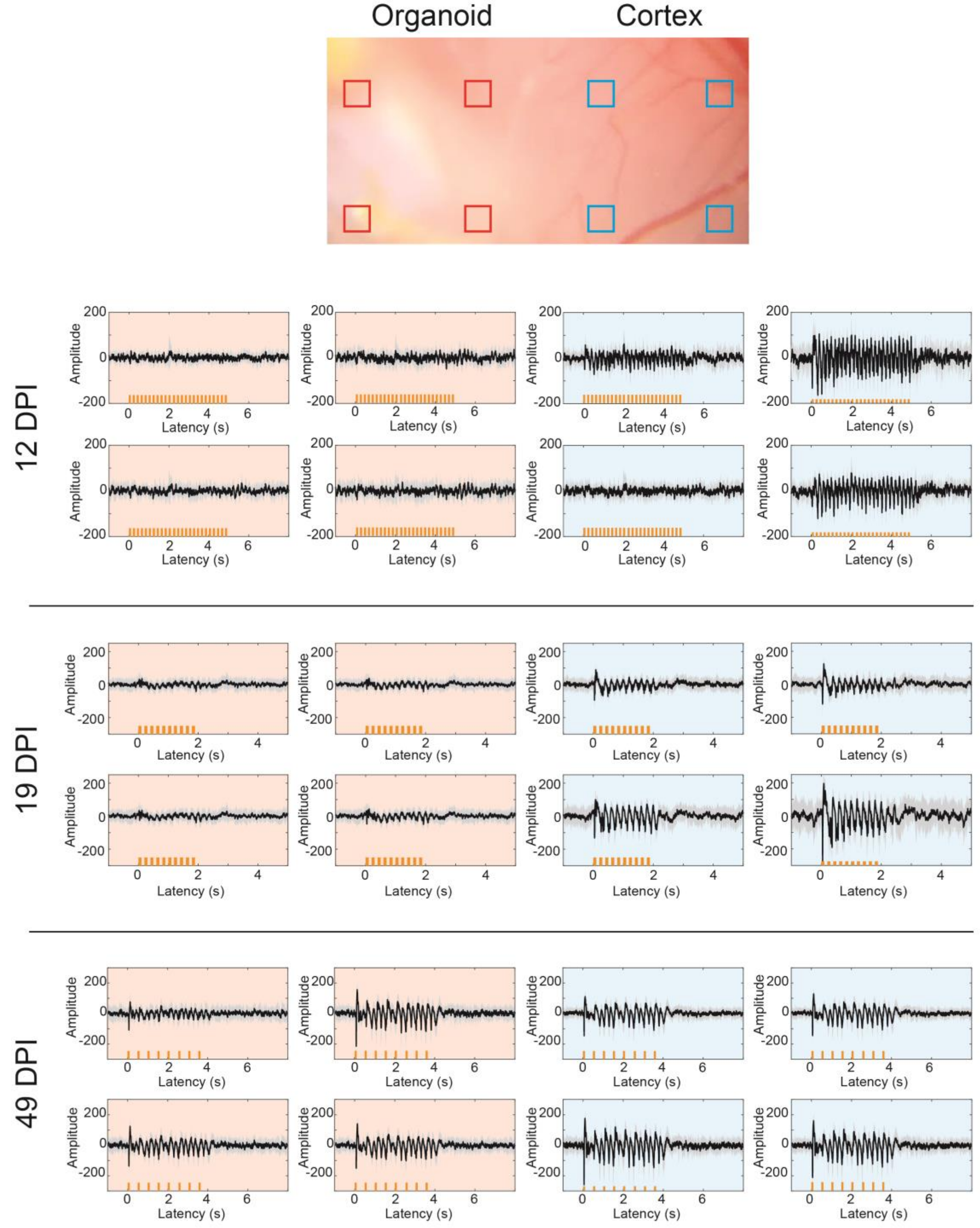

Supplementary Figure 2. Increase of local field potential (LFP) amplitude over time. LFP are shown as mean ± sdv (n=10 trials for 12 and 19 dpi and n=20 trials for 49 dpi). The brightfield

image shows the relative location of the channels shown; channels covering the organoid implantation area are outlined in red. The results shown are representative for a total of five animals.

Local field potential peak delay across channels

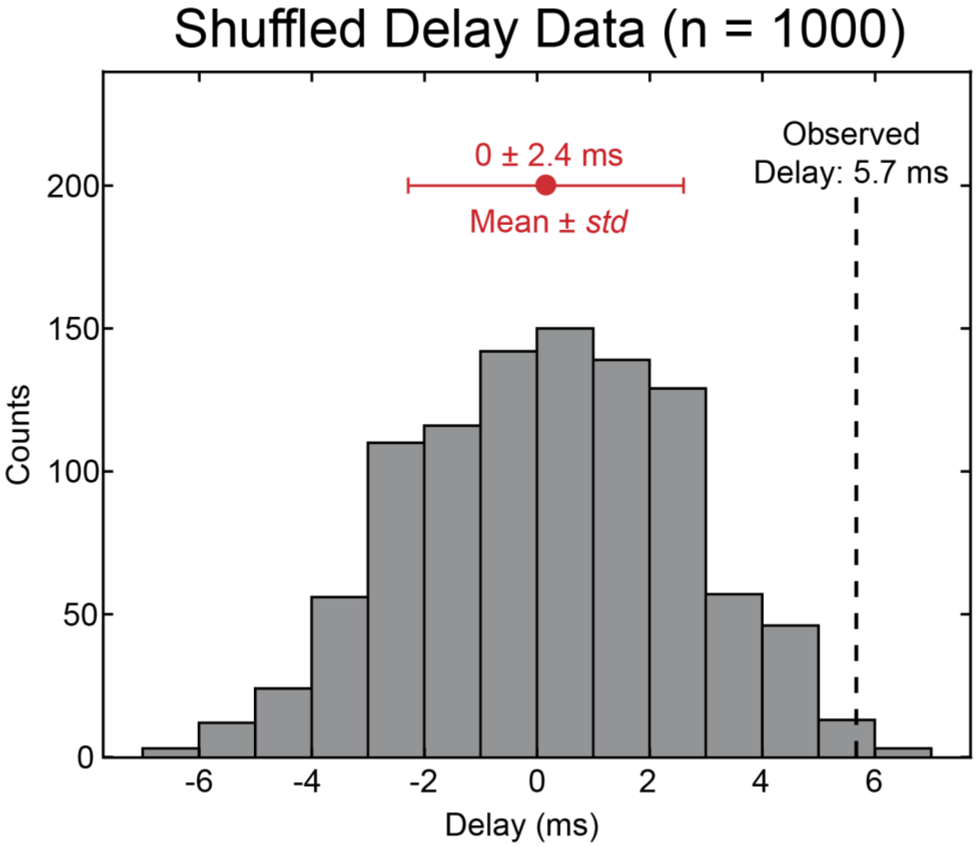

Supplementary Figure 3. Results of Student t test method of shuffling delay data between channels, calculating delays between organoid and cortex channel, and computing the p-value for our observed delay of 5.7 ms (p = 0, two-sided t test).

Low frequency spectrogram during stimuli

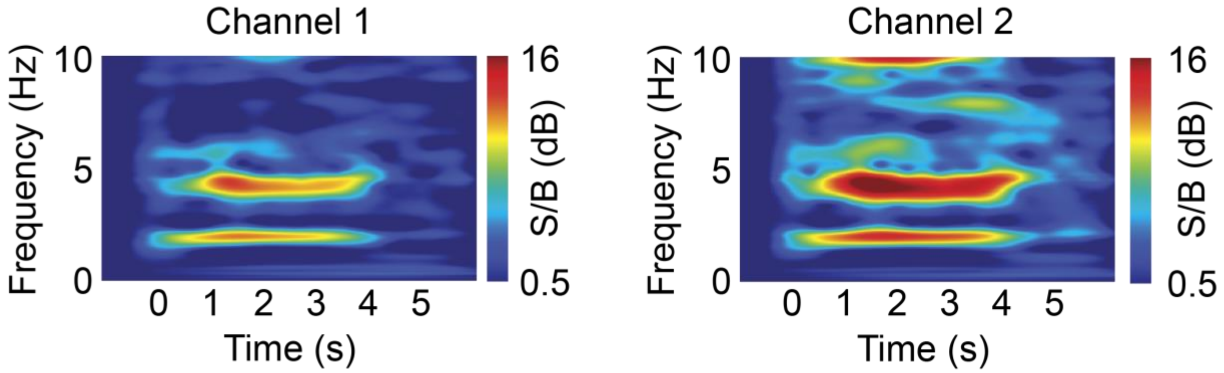

Supplementary Figure 4. Spectrograms (0-10 Hz) of the response to 2 Hz, 4 s visual stimulation. Channel 1 is overlaying organoid and Channel 2 is overlaying cortex. The recordings are the same as the ones shown in Figure 2e, acquired in a mouse 69 dpi.

MUA event rates in different mice

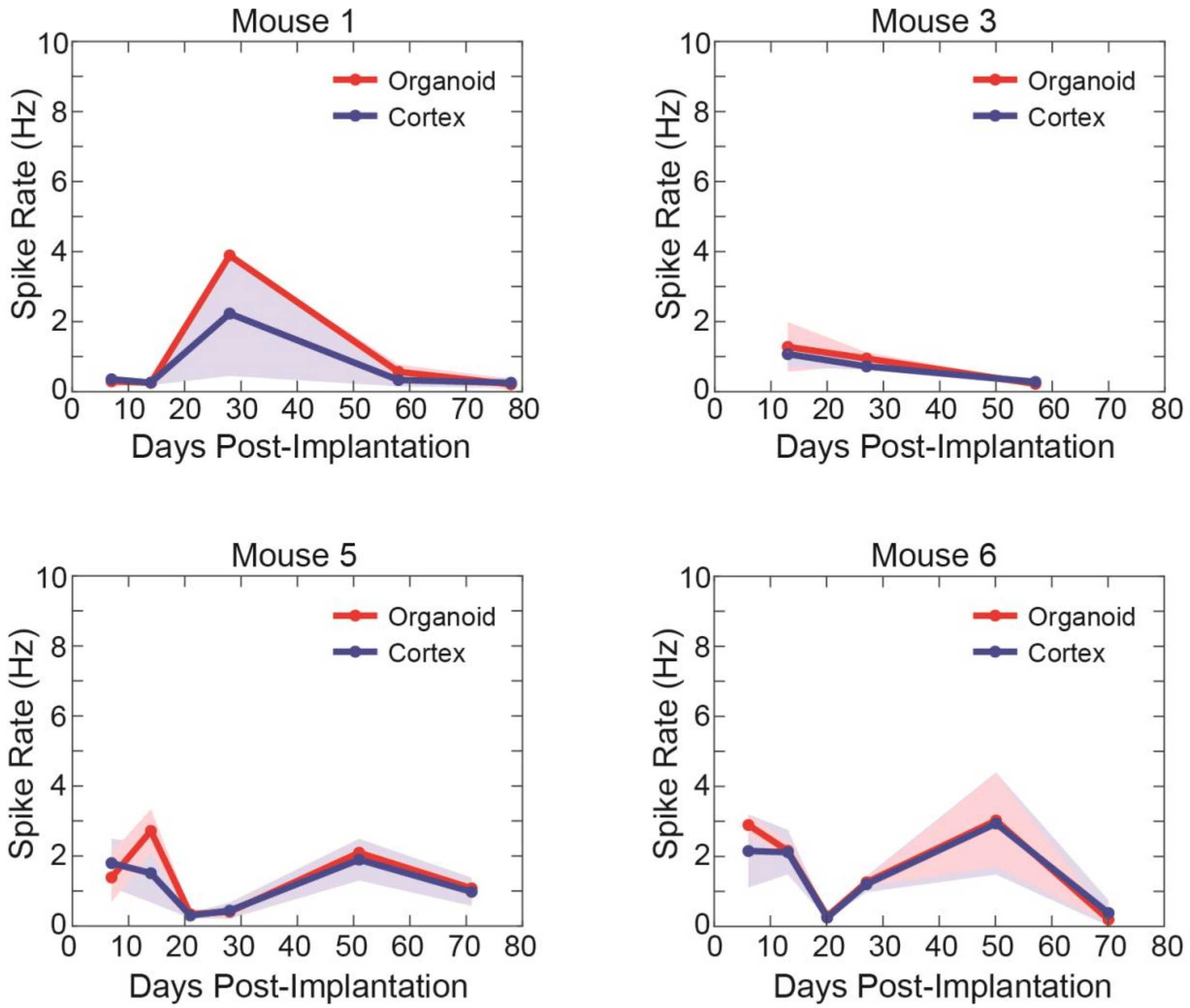

Supplementary Figure 5. Change in MUA spike rates over time for four mice. Rates are shown as mean ± sdv (of 5±3 channels for mouse 1 and 3 and 13±3 channels for mice 5 and 6) for channels overlaying cortex or organoid implantation. A MUA event threshold of -4*sdv was used for all recordings.

MUA event rates and raster

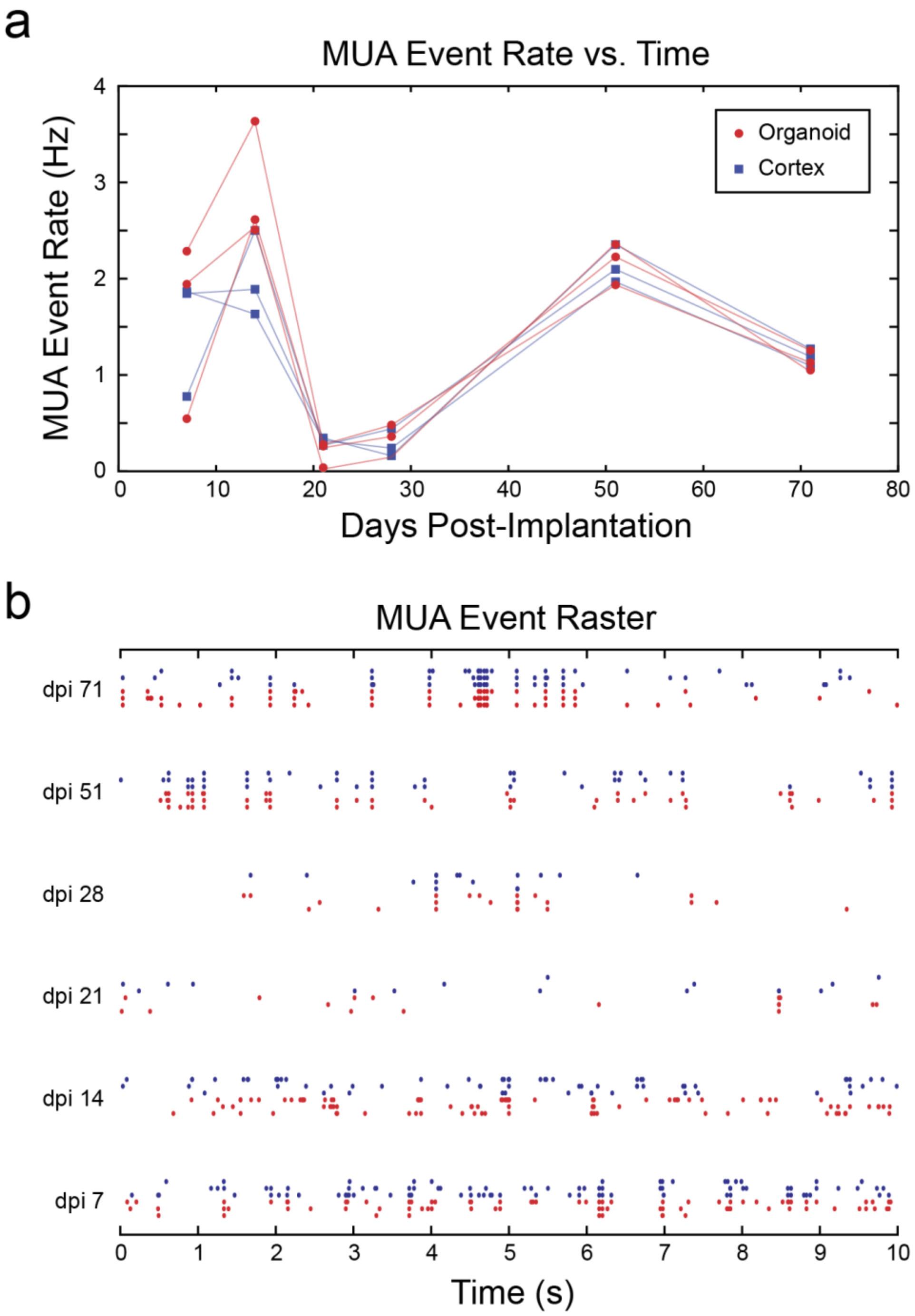

Supplementary Figure 6. (**a**) MUA event rates for three organoid and three cortex channels on recording days 7, 14, 21, 28, 51, and 71 post-implantation. (**b**) Raster plot of spontaneous MUA events in three organoid and three cortex channels.

MUA analysis

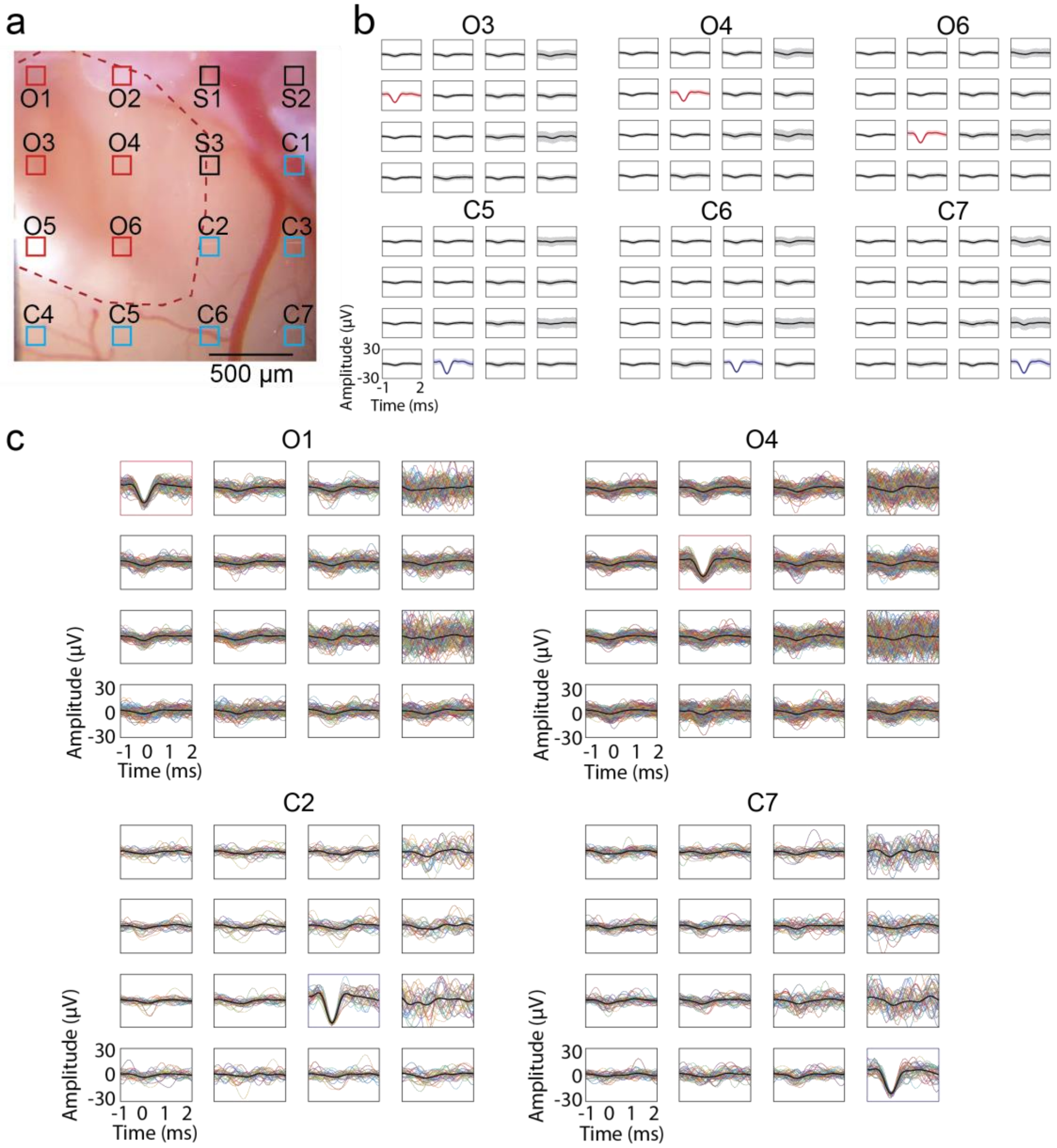

d

Ch	S1	S3	O2	O1	O3	O4	O5	C4	C5	O6	C6	C7	C3	C2	C1	S2
S1	147	1	2	2	0	1	2	1	1	0	1	0	0	0	0	0
S3	1	80	2	0	1	2	1	0	1	1	0	0	0	0	0	0
O2	2	2	118	0	1	1	2	0	1	0	1	0	0	0	0	0
O1	2	0	0	97	0	0	2	0	0	0	0	0	0	0	0	0
O3	0	1	1	0	127	1	1	1	0	0	0	0	0	0	0	0
O4	1	2	1	0	1	213	4	0	1	1	1	0	0	0	0	0
O5	2	1	2	2	1	4	139	0	1	0	1	3	0	0	0	0
C4	1	0	0	0	1	0	0	69	0	0	1	0	0	0	0	0
C5	1	1	1	0	0	1	1	0	55	0	1	0	0	0	0	0
O6	0	1	0	0	0	1	0	0	0	126	0	0	0	0	0	0
C6	1	0	1	0	0	1	1	1	1	0	97	0	0	0	0	0
C7	0	0	0	0	0	0	3	0	0	0	0	36	0	0	0	0
C3	0	0	0	0	0	0	0	0	0	0	0	0	4	0	0	0
C2	0	0	0	0	0	0	0	0	0	0	0	0	0	26	0	0
C1	0	0	0	0	0	0	0	0	0	0	0	0	0	0	30	0
S2	0	0	0	0	0	0	0	0	0	0	0	0	0	0	0	13

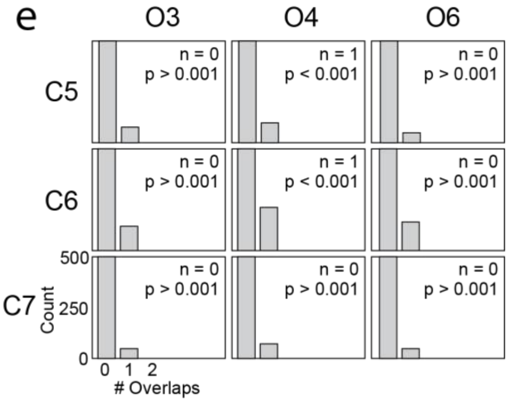

Supplementary Figure 7. MUA analysis in three mice to investigate the overlap of signal across channels. (**a**) Brightfield image of mouse cortex with organoid region outlined in red. Red channels are those overlapping the organoid. (**b**) Event-averaged MUA traces show that the MUA events are localized spatially (mean ± sdv). (**c**) Event-triggered MUA traces shown without averaging showing the waveforms in more detail. (**d**) Table showing the number of overlapping events after binning events into 1 ms windows for a ~100 s spontaneous recording trial. Diagonal shows the number of events detected per channel. Red color channels are channels overlaying the organoid, blue color channels are those overlaying cortex. (**e**) Histograms of the number of overlaps across an organoid channel and cortex channel (shown in plot titles) after circularly shuffling the MUA event trains 10,000 times. P-values were determined by integrating the shuffled counts from the overlap count of the non-shuffled case (n) in panel c to infinity (one-sided). Large p-values indicate no significant overlap across channels, supporting that the MUA data is independent across channels. Exact p-values are 0.0081 (O3 to C5), 0.0127 (O3 to C6), 0.0051 (O3 to C7), 0 (O4 to C5), 0.0002 (O4 to C6), 0.0077 (O4 to C7), 0.005 (O6 to C5), 0.0144 (O6 to C6), and 0.0053 (O6 to C7). The results shown are representative for a total of three animals.

Anesthetized vs. awake spectrograms

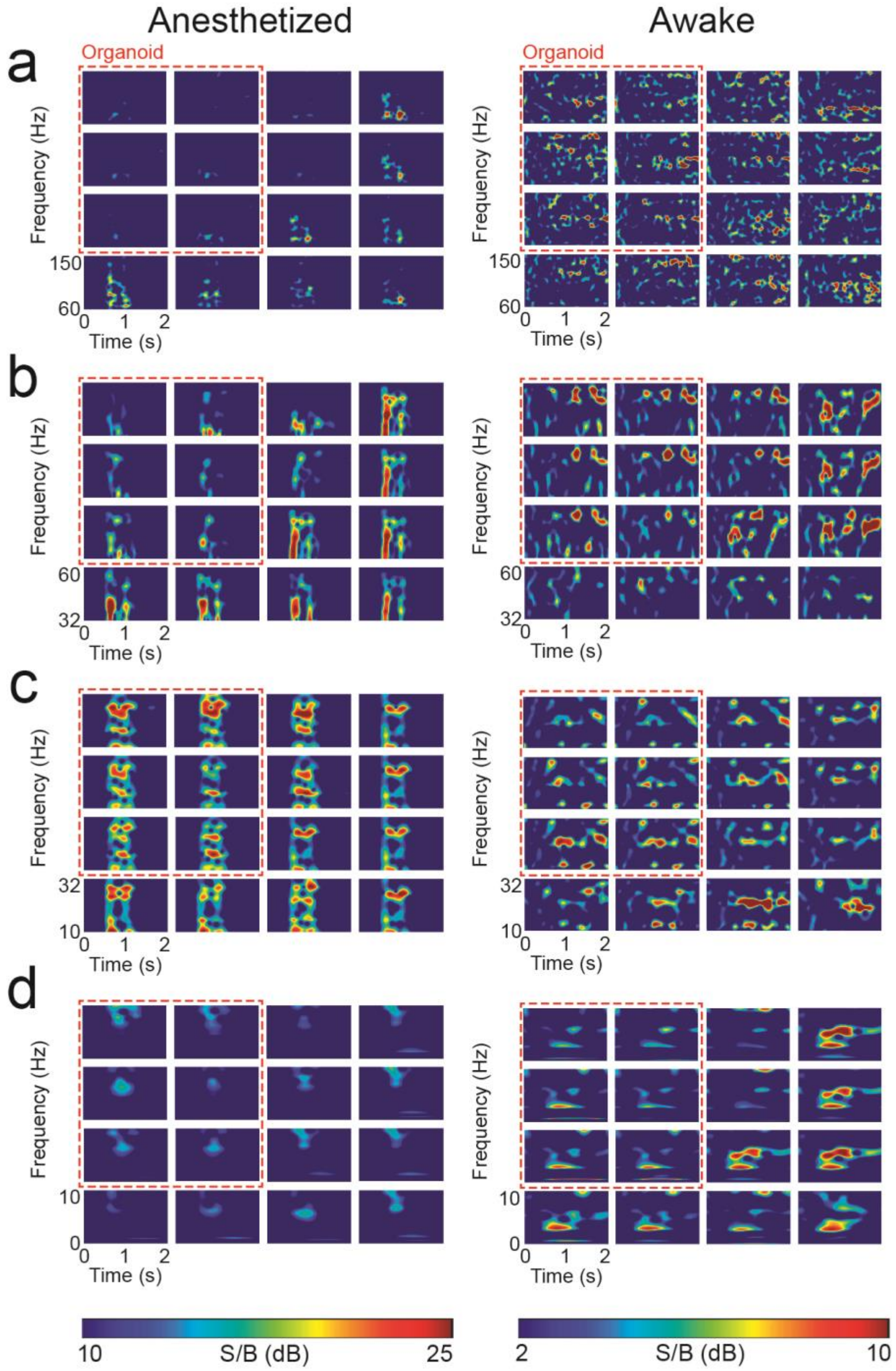

Supplementary Figure 8. Spectrograms during representative epochs (same epochs as figure 4c and 4e) for different frequency bands broken into sub-frequency ranges for easier visualization:

(**a**) 60-150 Hz, (**b**) 32-60 Hz, (**c**) 10-32 Hz, and (**d**) 0-10 Hz. The red dashed box in all panels delineates channels overlaying the organoid. A discrepancy appears along the organoid border for low and high (> 32 Hz) gamma bands while the mouse was under anesthesia with 1.5% isoflurane.

LFP amplitude during burst event under anesthesia

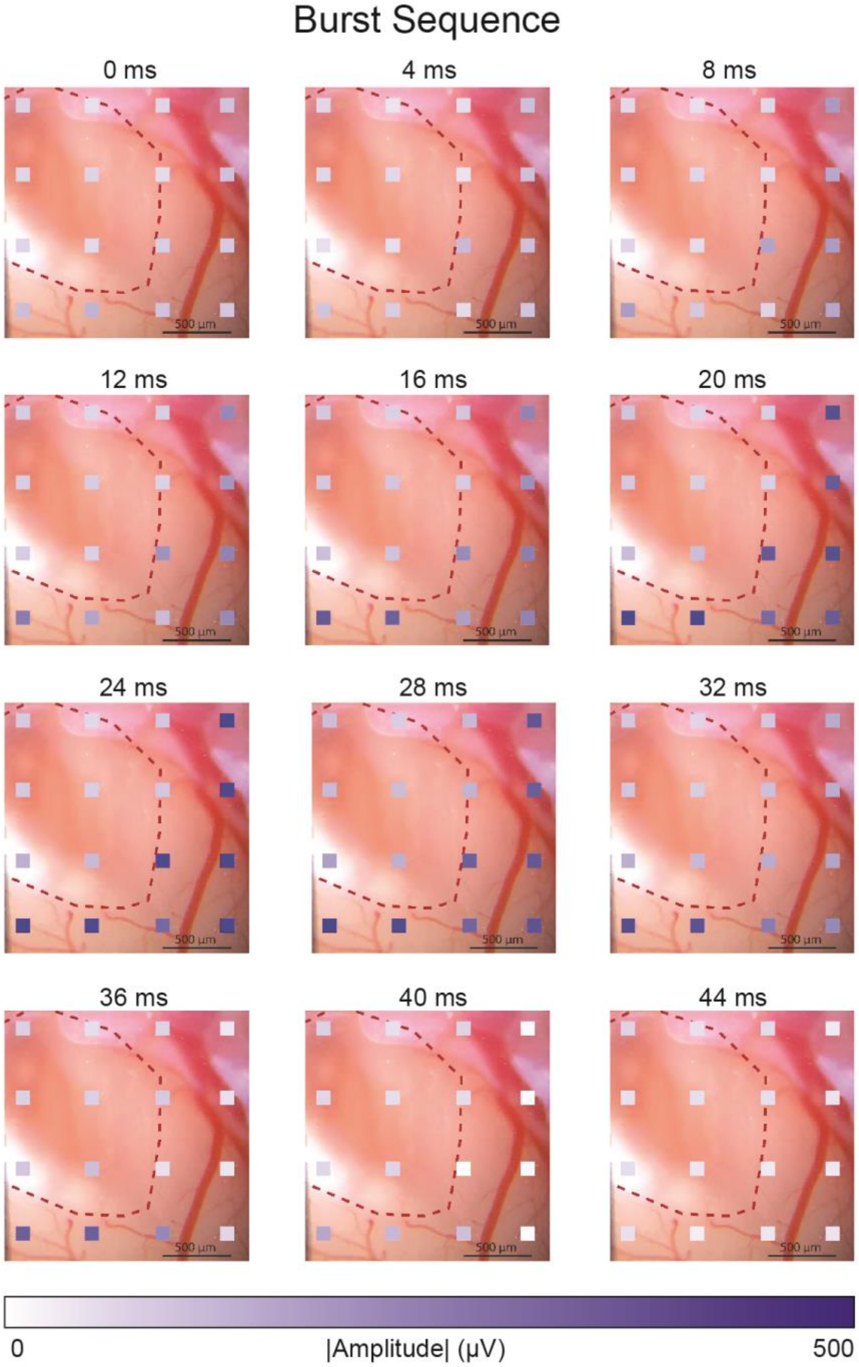

Supplementary Figure 9. Local field potential amplitude for all 16 channels during a burst event while the mouse was under anesthesia with 1.5% isoflurane. The results shown are representative for a total of five animals.

NM-95 traveling cells

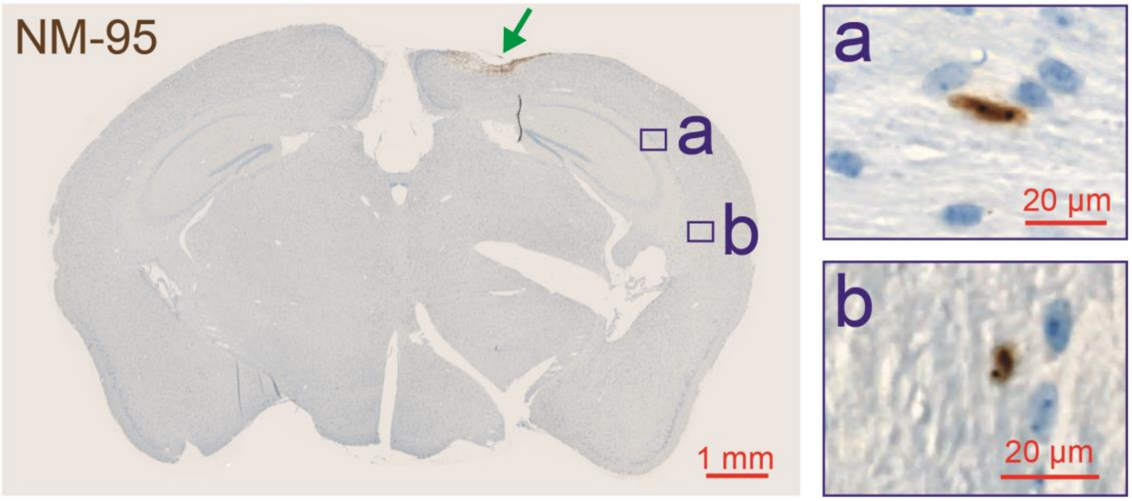

Supplementary Figure 10. Human (NM-95-positive) cells were observed at the implantation site (green arrow) and further away from the implantation site (**a, b**); we detected individual human cells along corpus callosum up to ~4 mm away from the implantation site (**b**). The results shown were repeated and are representative for a total of five animals.

Immunofluorescence for synaptophysin

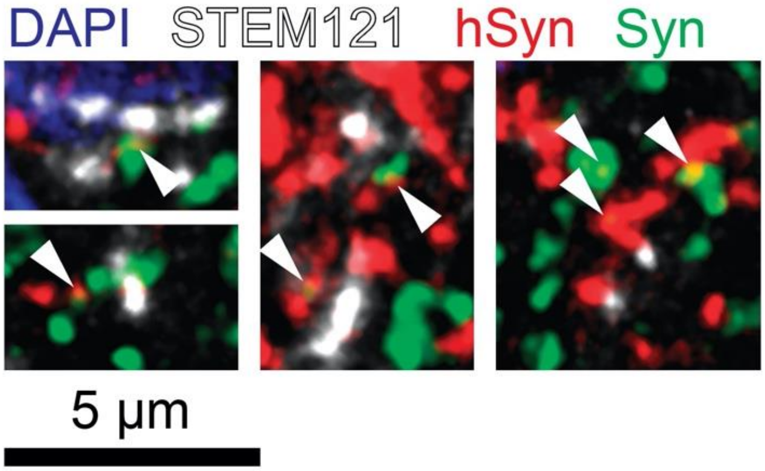

Supplementary Figure 11. Puncta that labeled positive for both Syn (green) and hSyn (red) were counted as presynaptic puncta of human origin (yellow, arrowheads). We observed a presence of STEM121 (white) and human presynaptic puncta (yellow, arrowheads) within regions of visual cortex, supporting the organoid extended axonal connections towards and into mouse visual cortex. The results shown were repeated and are representative for a total of two animals.

Comparison of Morlet wavelet and STFT methods

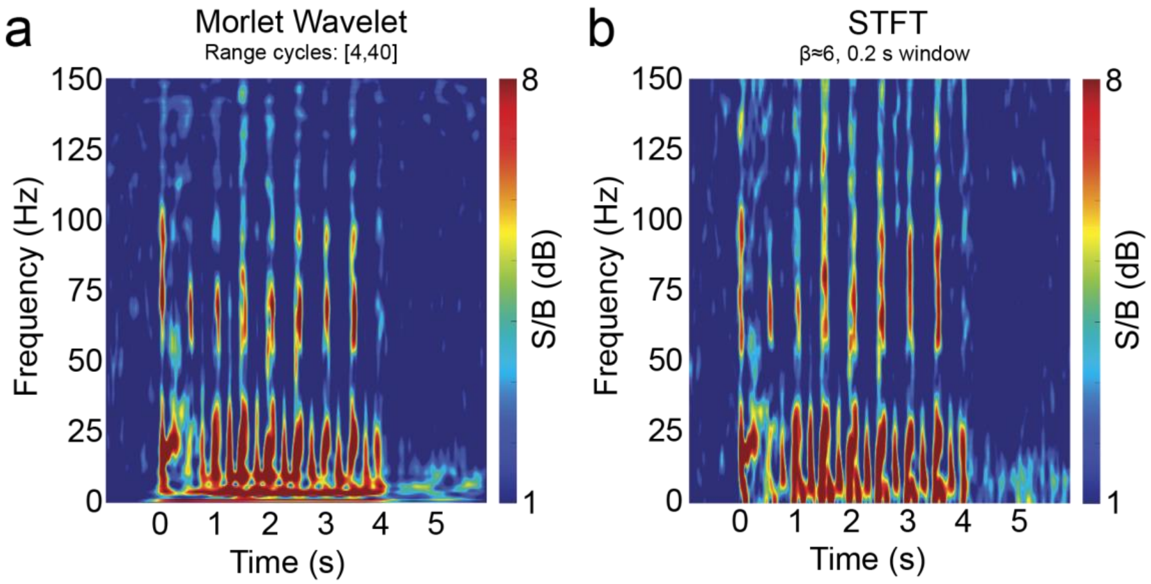

Supplementary Fig. 12. Spectrograms of the response to light stimuli generated using Morlet- (left) and Fourier- (right) based methods yield similar results. The Short-Time Fourier Transform (STFT) spectrogram was calculated using MATLAB's *pspectrum.m* function with a 0.2 s time window, 95% overlap, and leak value of 0.85, approximating a Hanning window.
