# Supplementary material for: Multimodal monitoring of human cortical organoids implanted in mice reveal functional connection with visual cortex
Source: Nat Commun. 2022 Dec 26;13:7945. doi: 10.1038/s41467-022-35536-3 (PMC9792589; doi:10.1038/s41467-022-35536-3)
Supplement: Supplementary file 3 — Description of Additional Supplementary Files [file 41467_2022_35536_MOESM3_ESM.pdf]

Description of Additional Supplementary Files

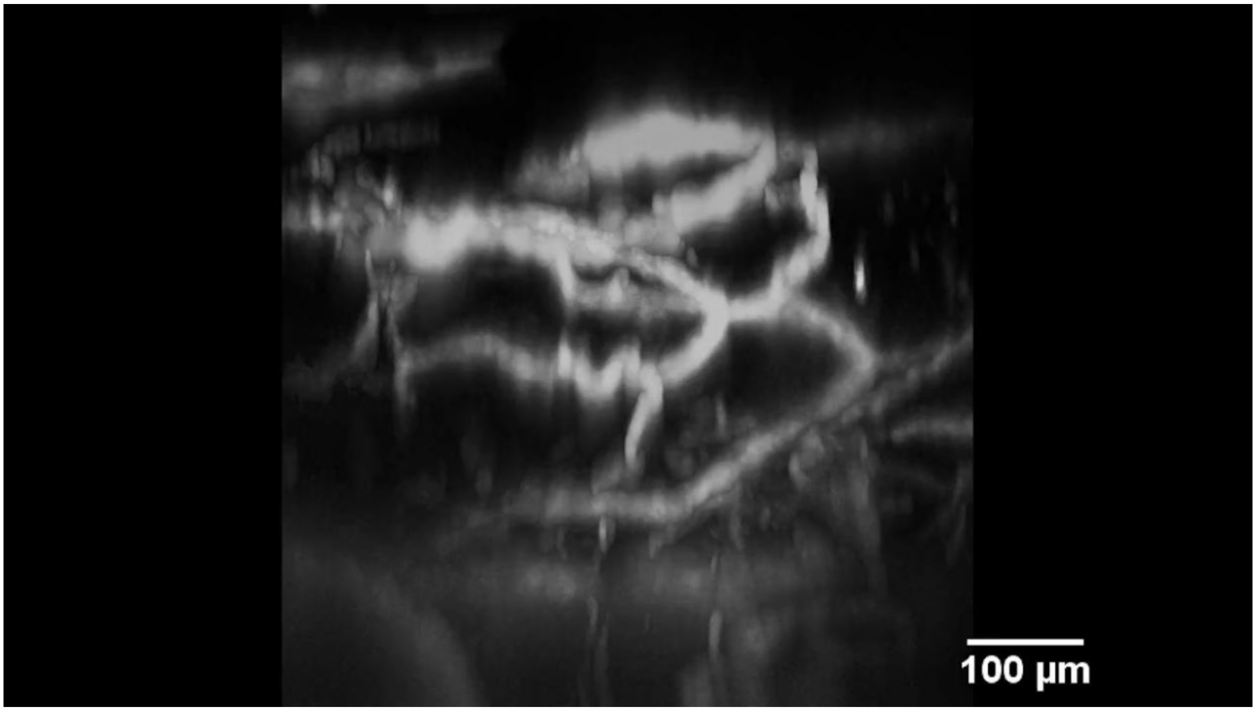

Supplementary Video 1 (screenshot). Three-dimensional projection of the vasculature within the organoid implant region. Image was acquired using two-photon microscopy after injection of Alexa Fluor 680 Dextran.
