# Supplementary material for: Multimodal monitoring of human cortical organoids implanted in mice reveal functional connection with visual cortex
Source: Nat Commun. 2022 Dec 26;13:7945. doi: 10.1038/s41467-022-35536-3 (PMC9792589; doi:10.1038/s41467-022-35536-3)
Supplement: Supplementary file 5 — Reporting Summary [file 41467_2022_35536_MOESM5_ESM.pdf]

nature portfolio

Corresponding author(s): Duygu Kuzum, Anna Devor

Last updated by author(s): Nov 28, 2022

Reporting Summary

Nature Portfolio wishes to improve the reproducibility of the work that we publish. This form provides structure for consistency and transparency in reporting. For further information on Nature Portfolio policies, see our Editorial Policies and the Editorial Policy Checklist.

Statistics

For all statistical analyses, confirm that the following items are present in the figure legend, table legend, main text, or Methods section.

n/a	Confirmed	
☐	☒	The exact sample size (*n*) for each experimental group/condition, given as a discrete number and unit of measurement
☐	☒	A statement on whether measurements were taken from distinct samples or whether the same sample was measured repeatedly
☐	☒	The statistical test(s) used AND whether they are one- or two-sided<br>*Only common tests should be described solely by name; describe more complex techniques in the Methods section.*
☒	☐	A description of all covariates tested
☒	☐	A description of any assumptions or corrections, such as tests of normality and adjustment for multiple comparisons
☐	☒	A full description of the statistical parameters including central tendency (e.g. means) or other basic estimates (e.g. regression coefficient) AND variation (e.g. standard deviation) or associated estimates of uncertainty (e.g. confidence intervals)
☒	☐	For null hypothesis testing, the test statistic (e.g. *F*, *t*, *r*) with confidence intervals, effect sizes, degrees of freedom and *P* value noted<br>*Give P values as exact values whenever suitable.*
☒	☐	For Bayesian analysis, information on the choice of priors and Markov chain Monte Carlo settings
☒	☐	For hierarchical and complex designs, identification of the appropriate level for tests and full reporting of outcomes
☒	☐	Estimates of effect sizes (e.g. Cohen's *d*, Pearson's *r*), indicating how they were calculated

Our web collection on statistics for biologists contains articles on many of the points above.

Software and code

Policy information about availability of computer code

Data collection	Two photon imaging was performed with an Ultima two-photon laser scanning microscopy system from Bruker Fluorescence Microscopy operated with PrairieView. Electrophysiology was collected using an Intan Technology RHD2000 amplifier board and RHD2000 evaluation system. Electrochemical Impedance Spectroscopy was performed using a Gamry Reference 600 Potentiostat. LED stimulation was delivered with a National Instruments DAQ system connected to the Intan amplifier board and controlled using custom MATLAB software.
Data analysis	Custom MATLAB (Mathworks, v2019b) code, built-in MATLAB functions, the Chronux toolbox (Bokil et al. 2010), and the Circular Statistics Toolbox for MATLAB (Berens 2021) were used for data analysis. Algorthms used include: independent component analysis using the jadeR alforithm from EEGLab (Delorme and Makeig 2004; Delorme, Sejnowski, and Makeig 2007; Stejnowski 1996; Jung 1998), filtering using a Chebyshev filter (designfilt.m, filtfilt.m), Morlet wavelet spectral analysis, peak detection (findpeaks.m), Student's t test (ttest.m, ttest2.m), multitaper method (modified function mtspecgramc.m from the Chronux toolbox), phase locking values, and bootstrap sampling method. The code used for processing neural recordings are available at: https ://github.com/mwilson UCSD/multimodal_ organoids. FIJI (ImageJ 1.53k, Java l.8.0_172, 64-bit for Windows), Leica Microsystems LAS AF Lite (Version 2.6.0 build 7266), and ZEN 3.2 (blue edition, Carl Zeiss Microscopy GmbH) were used for image analysis. Adobe Illustrator (25.2.3, 64-bit) was used for figure preparation.

For manuscripts utilizing custom algorithms or software that are central to the research but not yet described in published literature, software must be made available to editors and reviewers. We strongly encourage code deposition in a community repository (e.g. GitHub). See the Nature Portfolio guidelines for submitting code & software for further information.

Data

Policy information about availability of data

All manuscripts must include a data availability statement. This statement should provide the following information, where applicable:

- Accession codes, unique identifiers, or web links for publicly available datasets
- A description of any restrictions on data availability
- For clinical datasets or third party data, please ensure that the statement adheres to our policy

The raw data that support the findings of this study are available on request from the corresponding authors. Source data with quantifications for tables, charts, and statistics are provided with this paper in the Source Data file.

Human research participants

Policy information about studies involving human research participants and Sex and Gender in Research.

Reporting on sex and gender	N/A
Population characteristics	N/A
Recruitment	N/A
Ethics oversight	N/A

Note that full information on the approval of the study protocol must also be provided in the manuscript.

Field-specific reporting

Please select the one below that is the best fit for your research. If you are not sure, read the appropriate sections before making your selection.

☒ Life sciences ☐ Behavioural & social sciences ☐ Ecological, evolutionary & environmental sciences

For a reference copy of the document with all sections, see nature.com/documents/nr-reporting-summary-flat.pdf

Life sciences study design

All studies must disclose on these points even when the disclosure is negative.

Sample size	Sample size of mice with implanted organoids was determined empirically based on previous study sample sizes (Mansour, A. A., J. T. Goncalves, C. W. Bloyd, H. Li, S. Fernandes, D. Quang, S. Johnston, S. L. Parylak, X. Jin, and F. H. Gage. 2018. 'An in vivo model of functional and vascularized human brain organoids', Nat Biotechnol, 36: 432-41.) and aimed to use the minimum number of live animals.
Data exclusions	Any electrophysiology data containing noise from mouse movement (detected using a video camera) was excluded from analysis.
Replication	Results were verified between animals; one batch of animals was used for this study due to external timelines.
Randomization	No randomization was used in this study. All mice received organoid implants and were subject to the same conditions and tests.
Blinding	Experimenters were not blind to the culture or grafting of organoids nor to electrophysiology, imaging, or histology because all mice and organoids received the same treatment.

Reporting for specific materials, systems and methods

We require information from authors about some types of materials, experimental systems and methods used in many studies. Here, indicate whether each material, system or method listed is relevant to your study. If you are not sure if a list item applies to your research, read the appropriate section before selecting a response.

Materials & experimental systems

n/a	Involved in the study	
☐	☒	Antibodies
☐	☒	Eukaryotic cell lines
☒	☐	Palaeontology and archaeology
☐	☒	Animals and other organisms
☒	☐	Clinical data
☒	☐	Dual use research of concern

Methods

n/a	Involved in the study	
☒	☐	ChIP-seq
☒	☐	Flow cytometry
☒	☐	MRI-based neuroimaging

Antibodies

Antibodies used

NM-95/human nucleoli (1:300, Abeam ab190710), NeuN (1:300, EMD Millipore), and CD31 (1:300, Dianova), STEM121 (1:75, Takara Y40410), hSyn (1:750, lnvitrogen 14-6525-80), total synaptophysin (1:3000, lnvitrogen MAl-213), rabbit anti-Ms (IgG1, IgG2a, IgG3; Abcam ab133469; 1:1000), tertiary HRP polymer-linked anti-Rb (OmniMap; 05266548001; Ventana Medical systems).

Validation

NM95/Human nucleoli: This antibody was validated in human neurosurgical FFPE tissue as well as a mouse xenograft model where human glial cells were grafted into the mouse brain to confirm the specificity in a mixed-species setting. The manufacturer website confirms validation in FFPE tissue and lists 9 publications while CiteAb has 20 for this antibody.

NeuN: This antibody was validated in human neurosurgical FFPE tissue and mouse FFPE tissue. The manufacturer website confirms this antibody recognizes a wide range of species for NeuN. Both manufacturer and CiteAb list over one hundred publications using this antibody.

CD31: This antibody has been validated in various tissue types and all show strong vessel staining. The manufacturer website indicates it is validated for FFPE, has dozens of images in various tissue types and lists over 30 publications while CiteAb lists over 300 publications.

STEM121: This antibody was validated in human neurosurgical FFPE tissue. The manufacturer website indicates this protein “reacts specifically with a cytoplasmic protein of human cells. This marker is expressed in cells from a variety of tissues including brain, liver and pancreas. However, it is expressed most highly in central nervous system (CNS) cells. This antibody does not cross-react with brain tissue or extracts from mouse, rat, or cynomologous monkey.” The manufacturer website lists over 90 publications while CiteAb currently doesn’t have a listing for this antibody.

hSynaptophysin: This antibody was validated in human neurosurgical FFPE tissue. The manufacturer website confirms the antibody is validated for use in FFPE tissue, has images indicating the staining pattern, but does not list any publications. CiteAb lists 6 citations.

Total synaptophysin: This antibody was validated in human neurosurgical FFPE tissue. The manufacturer website confirms validation of this antibody in FFPE tissue, has 6 appropriate images, and lists 6 citations as does CiteAb.

Eukaryotic cell lines

Policy information about cell lines and Sex and Gender in Research

Cell line source(s)

iPSC lines derived from control individuals as described in Trujillo et al. 2019.

Authentication

See Trujillo et al. 2019.

Mycoplasma contamination

See Trujillo et al. 2019.

Commonly misidentified lines
(See ICLAC register)

N/A

Animals and other research organisms

Policy information about studies involving animals; ARRIVE guidelines recommended for reporting animal research, and Sex and Gender in Research

Laboratory animals

Eight immune-deficient non-obese diabetic (NOD)/severe combined immunodeficient (SCIO) female mice aged 6 to 8 weeks old were acquired from Jackson Laboratories (JAX Stock: 001303). Animals were kept in autoclaved cages under standard conditions (20-22 °C, 40-60% relative humidity) on a 12 h light/dark cycle with ad libitum access to food and water.

Wild animals

Study did not involve wild animals.

Reporting on sex

Eight female mice were used in this study. Differences in sex were not considered for this study.

Field-collected samples

Study did not involve field-collected samples.

Ethics oversight	Study was conducted in accordance with the National Institutes of Health's Guide for the Care and Use of Laboratory Animals and was approved by University of California San Diego's Institutional Animal Care and Use Committee (IACUC) under protocol S14275.

Note that full information on the approval of the study protocol must also be provided in the manuscript.
